# Transthoracic echocardiographic assessment of IVC diameter variability to determine fluid responsiveness in children with septic shock: a pilot study

**DOI:** 10.1186/cc11773

**Published:** 2012-11-14

**Authors:** K Sasidaran, M Jaishree, S Singhi, R Manoj

**Affiliations:** 1 PGIMER, Chandigarh, India

## Background

Hemodynamic monitoring plays a key role in the early recognition, optimization of interventions and monitoring of therapeutic response in children with septic shock. Assessing the need for fluids followed by rapid and timely fluid resuscitation is crucial for improved outcomes. It is in this context that fluid responsiveness, defined as an increase in cardiac output in response to a fluid challenge, assumes importance. We have done this prospective clinical study to evaluate the degree of IVC diameter variability in predicting fluid responsiveness (increment in stroke index ≥15%) in children with septic shock post 20 ml/kg of crystalloid (0.9% saline) resuscitation.

## Methods

A total of 166 episodes of preload responsiveness check were echocardiographically evaluated in 41 children with septic shock. In each episode, IVC diameter variability ((maximum - minimum IVC diameter)/maximum IVC diameter), stroke index and ejection fraction were assessed at two points (before preload T0 and after preload T1). Adequate sedation was ensured before each echocardiographic assessment. Infants <1 month of completed age, any clinical evidence of increased intra-abdominal pressure, children with previously diagnosed heart disease, requiring high-frequency ventilation, and for whom family did not give consent were excluded from the study.

## Results

Of the 166 episodes, 120 (72%) were fluid responsive and 46 (28%) were nonresponsive. One hundred and twenty-five episodes occurred on patients who were on positive pressure ventilation, whilst 41 occurred during spontaneous breathing. The decrease in the heart rate with preload was significant in the responsive as compared with the nonresponsive group (24 ± 9 vs. 4 ± 7; *P *= 0.001) independent of the ventilator support. IVC diameter variability at T0 correlated significantly (*r *= 0.39; *P *= 0.001) with stroke index increment following preload. The AUC of ROC for IVC diameter variability was 0.75 (0.66 to 0.85). A cutoff value of 14% variability showed 84.4% sensitivity and 65.9% specificity to positively predict fluid responsiveness in ventilated as well in spontaneously breathing children.

## Conclusion

IVC diameter variability showed a significant correlation with stroke index increment after preload and can act as a useful bedside tool in predicting preload responsiveness in children with septic shock. Utility of a serial ΔIVC percentage instead of a single measurement to assess volume changes needs to be explored.

